# Association between nutrition knowledge and Mediterranean diet adherence in Israeli adults with type 2 diabetes

**DOI:** 10.1038/s41430-026-01781-8

**Published:** 2026-07-01

**Authors:** Michal Kasher Meron, Adi Givati, Muhamad Agbaria, Liat Barzilay Yoseph, Sofia Shapira, Erez Ramati, Nuha Younis Zeidan, Vered Kaufman-Shriqui, Ofra Kalter–Leibovici, Pnina Rotman-Pikielny

**Affiliations:** 1 Department of Endocrinology, Meir Medical Centre, Kfar Saba, Israel; 2 School of Medicine, Gray Faculty of Medical and Health Sciences, Tel-Aviv University, Tel-Aviv, Israel; 3 Internal Medicine Department, Meir Medical Centre, Kfar Saba, Israel; 4 Meuhedet Health Services, Tel Aviv, Israel; 5 Clalit Health Services, Tel Aviv, Israel; 6 Department of Nutrition Sciences, Ariel University, Ariel, Israel; 7 Dina Recanati School of Medicine, Reichman University, Herzliya, Israel; 8 Gertner Institute of Epidemiology and Health Policy Research, Sheba Medical Centre, Ramat Gan, Israel

**Keywords:** Nutrition, Lifestyle modification, Patient education

## Abstract

**Objectives:**

To investigate the association between dietary knowledge and adherence to Mediterranean diet (MedD) among people with type 2 diabetes (T2D) in Israel.

**Methods:**

In this cross-sectional single-center study, adults with T2D visiting a diabetes clinic were enrolled for an interviewer-administered survey. Dietary knowledge was assessed using a questionnaire adapted for locally available foods. MedD adherence was measured using the Israeli Mediterranean Diet Adherence Screener (I-MEDAS). Additional clinical and anthropometric characteristics were retrieved from patients’ medical records. The association between dietary knowledge and MedD adherence was analyzed in a multivariable logistic model.

**ResultsF:**

A total of 134 people were included. Mean age was 70 ± 10.7 years; 54.5% were women. The mean dietary knowledge score was 12.1 ± 2.4 (range: 0–17), and the MedD adherence score was 11.0 ± 2.1 (range: 0–17). In multivariable analysis, adjusted for age, sex, socio-economic status and diabetes duration, people in the highest tertile of dietary knowledge were more likely to be in the highest tertile of MedD adherence (adjusted odds ratio 4.6, 95% CI 1.5–14.8, *p* = 0.01), compared to those in the lowest knowledge tertile. The model explained limited variance in adherence (Nagelkerke R² = 0.16). Sensitivity analyses using the exposure and outcome variables as continuous scores were not statistically significant.

**Conclusion:**

Dietary knowledge showed a modest association with MedD adherence in Israeli adults with type 2 diabetes, but this association lacked robustness across analytical approaches. Dietary knowledge explains a small proportion of dietary adherence variability, suggesting that unmeasured factors play a more substantial role in determining dietary behavior.

## Introduction

Diet and nutrition are keystones of T2D management. Nutritional therapy has been shown to reduce hemoglobin A1C by up to 2% [1, 2], reduce the need for glucose-lowering medications, lower blood pressure and lipid levels, and may reduce the risk for diabetic complications [1, 3–5]. Guidelines for diabetes management across different countries underscore the importance of nutritional therapy in people with T2D [6–8]. They recommend an individualized approach, with no single ideal diet, although certain dietary patterns are more recommended than others. The MedD is one of the recommended eating patterns. It is based on intake of plant foods, olive oil, whole grain cereals, nuts and seeds, legumes, fish and sea food, and a moderate intake of dairy products. In randomized controlled trials, the MedD was shown to reduce the risk of major cardiovascular events in people with high cardiovascular risk, including people with diabetes [9–11].

Patient adherence to medical nutrition therapy poses a significant clinical challenge. Despite strong evidence supporting the MedD for T2D management [11], adherence remains suboptimal even in Mediterranean-adjacent regions such as Turkey, Spain, and Morocco, based on self-reporting questionnaires indicating low-to-moderate adherence rates [12]. Medical nutrition therapy aims to educate patients and promote healthy eating patterns [13], yet the relationship between diabetes-specific nutrition knowledge and MedD adherence has not been systematically examined in Middle Eastern populations.

In Israel, the MedD is endorsed by the Israeli Ministry of Health [14] and aligns with local food culture and traditions. This study investigates the association between patient dietary knowledge and MedD adherence in Israeli adults with T2D. Understanding this relationship may inform the planning of diabetes counseling programs in this population.

## Subjects and methods

### Study design and population

This cross-sectional study was conducted at a single diabetes clinic at Meir Medical Center, which is part of Clalit Health Services. It is a tertiary medical center serving approximately 1 million residents of a mainly urban area.

The inclusion criteria were age >18 years and a diagnosis of T2D. People with diabetes who were diagnosed less than six months prior to the diabetes clinic visit, pregnant women and people who were unable to converse in Hebrew or provide informed consent were excluded. Participants were recruited from those attending the diabetes clinic on randomly selected days between July 2022 and May 2023. Based on a sample size calculation to detect a correlation coefficient of 0.25 representing a moderate-to-large effect in behavioral research [15, 16], with type-I error of 5% and power of 80%, we determined that 123 participants were required. To account for potential data incompleteness, we aimed to recruit 134 participants (~9% above target). Of those we approached, 134 participants (~80%) agreed to participate and completed a 30-min interview with a trained interviewer. The remaining individuals either declined participation or could not be interviewed due to clinic scheduling constraints and interviewer availability. The study protocol was approved by the Meir Medical Centre Ethics Committee (MMC-0076-22).

### Study variables

The exposure variable was the participant’s dietary knowledge. Several instruments assess diabetes knowledge, incorporating dietary knowledge as one component of self-care practices [17–19]. Using these instruments often requires adaptations in language and cultural context especially for their dietary knowledge component. At the time of the study, there was no validated questionnaire in Hebrew specifically assessing diabetes-related dietary knowledge. Therefore, we used and adapted the dietary component of the Diabetes in the Arab Population in Israel (DAPI) questionnaire [20], which had been previously developed and validated in Israeli Arab populations with type 2 diabetes. Using closed-ended questions, the participants were asked to state whether several common food items “can be consumed freely”, “can be consumed in moderation” or “can be consumed only in rare situations (such as hypoglycemia)”. To make the questionnaire suitable for both Arab and Jewish Israeli populations, we made targeted cultural adaptations while maintaining the core construct—knowledge of appropriate food choices for diabetes management. Specific changes included: (1) replacing Arab-specific food items with culturally neutral equivalents from the same food category—for example, “dates” and “grapes” (both high in natural sugars), “bulgur or frike” with “whole grains”, and “biscuits and baked cookies” with “sweet pastries”; (2) expanding the item “rice” to “white rice or white bread or pita” to reflect staple carbohydrate sources consumed by both populations; and (3) adding questions about commercially packaged foods labeled for people with diabetes and preferred fat sources to align with current nutrition recommendations and the Israeli food environment. These modifications ensured the questionnaire was culturally relevant to both populations while preserving the assessment of knowledge across the same nutritional domains as the original DAPI.

The adapted questionnaire underwent two validation steps. First, a multidisciplinary team of endocrinologists, nutritionists, and public health specialists assessed face validity to ensure clarity and appropriateness. Second, we established content validity by individually presenting the questionnaire to 9 registered nutritionists specialized in diabetes care, working in hospital or community settings. As registered nutritionists are the main providers of diabetes nutrition education in Israel, they were well-positioned to determine clinically relevant content and correct responses. Items were retained if at least 75% of nutritionists agreed on the correct answer [21, 22]. For instance, if over 75% of the nutritionists indicated that Coca-Cola beverage should be consumed ‘only in rare cases, such as hypoglycemia,’ this was the designated correct answer (worth one point) and the item was retained. Because this was a cultural adaptation of an existing validated instrument rather than de novo scale development, full psychometric validation was not conducted. The final version included 17 items. Internal consistency was assessed using Cronbach’s alpha (α = 0.52).

The main outcome variable was MedD adherence, measured by the 17-item I-MEDAS questionnaire [23], an adaptation of the 14-item MEDAS questionnaire from the Spanish PREDIMED study [24, 25]. In the original MEDAS questionnaire, participants were asked to estimate their daily or weekly intake of various foods, earning 1 point per item if predefined criteria were met. For instance, two servings or more of non-starchy vegetables servings per day, with 1 serving defined as 200 g, earned 1 point). The I-MEDAS includes food items that are in accordance with the MedD principles and are widely consumed in Israel (e.g., tahini, hummus and low-fat dairy) instead of food items that appeared in the original MEDAS and are rarely consumed locally (e.g., shellfish and savory tomato sauce). The I-MEDAS has been previously validated and shown to correlate with all-cause mortality in a large Israeli cohort study [23].

The participants’ electronic medical records were thoroughly reviewed to collect socio-demographic, clinical, biochemical, and administrative data. Routine laboratory tests taken up to 6 months prior to the interview visit were recorded. Information on macrovascular and microvascular diabetes complications was based on documented diagnoses or laboratory tests. Nephropathy was considered present if noted by the clinician or evident based on lab tests of serum creatinine level ≥1.3 mg/dL or urine albumin/creatinine ratio >30. Area-based socioeconomic status was determined using the Israeli Socioeconomic Score (1–10), assigned based on participants’ residential address and periodically published by the Central Bureau of Statistics [26], where higher scores indicate greater affluence. This ecological index incorporates neighborhood-level indicators including average income, education level, employment rate, standard of living, and demographic characteristics.

### Statistical analysis

In this cohort, both dietary knowledge and MedD adherence scores were relatively high with limited variability. Therefore, dietary knowledge was categorized into tertiles, while MedD adherence was dichotomized into the highest tertile versus the lower two tertiles, to better capture potential differences in the “high-score” subgroup while still retaining a sufficiently large comparison group.

Baseline characteristics across dietary knowledge tertiles are presented as means and standard deviations for continuous variables and as frequencies and percentages for categorical variables. For categorical variables, Chi-square tests were used. For continuous variables, normality was assessed; normally distributed variables were compared using one-way ANOVA, while non-normally distributed variables were analyzed using the Kruskal–Wallis test.

The primary analysis used multivariable logistic regression to assess the association between dietary knowledge tertiles, using the knowledge lowest tertile as a reference, and high MedD adherence (upper tertile of MedD adherence, dichotomic variable), with adjustment for age, sex, duration of diabetes and socio-economic status. Odds ratios (OR) were calculated with 95% confidence intervals (CIs). Logistic regression assumptions were verified, including absence of multicollinearity among predictors (variance inflation factor <2.0 for all variables). The goodness of fit of the model was evaluated with the Hosmer–Lemeshow statistic and Nagelkerke R².

For descriptive purposes, the unadjusted association between dietary knowledge and MedD adherence tertiles was assessed using chi-square test.

Two sensitivity analyses were conducted. First, the primary logistic regression model was further adjusted to additional clinical variables, including BMI ≥ 30 kg/m², HbA1c, number of hypoglycemic agents, and number of diabetic complications. Second, linear regression was used with the outcome variable of MedD adherence defined as a continuous score, adjusted for the same covariates as in the main model. Data were analyzed using IBM SPSS statistics software version 29.0 (SPSS Inc., Chicago, IL, U.S.A.). A two-sided *P*-value < 0.05 was considered statistically significant.

## Results

A total of 134 people were included in the study. Their clinical and socio-demographic characteristics according to tertiles of the dietary knowledge score are presented in Table 1. The participants’ mean age was 70 ± 10.7 years. 54.5% of the participants were women, 96% were Jewish, and had a high socio-economic background, with a mean score of 7.8 out of 10. Most had diabetes for more than 15 years, with more than one target organ complication. Their serum glucose levels, hemoglobin A1C, LDL cholesterol levels, and blood pressure were relatively well controlled, with mean values of 132.8 ± 41.6 mg/dL, 7.1 ± 1.1%, 68.7 ± 25.0 mg/dL, and 137/72 mmHg, respectively.

**Table 1:** Study cohort characteristics by tertiles of dietary knowledge score.

Characteristic	Total *N* = 134 (100%)	Tertile 1 Score ≤ 11 *N* = 48 (35.8%)	Tertile 2 Score 12–13 *N* = 47 (35.1%)	Tertile 3 Score ≥ 14 *N* = 39 (29.1%)	*p*-value
Age, years	70 ±10.7	70.6 ±10.1	69.5 ±10.7	69.3 ±11.8	0.67
Male sex, *n* (%)	61 (45.5)	26 (54.2)	18 (38.3)	17 (43.6)	0.29
Sector, Jewish, *n* (%)	129 (96.3)	46 (95.8)	45 (95.7)	38 (97.4)	0.90
Socio-economic status, point^a^	7.8 ±1.1	7.8 ±1.3	7.8 ±1.0	7.9 ±1.1	0.68
Current cigarette smoker, yes, *n* (%)	11 (8.2)	8 (16.7)	10 (21.3)	7 (17.9)	0.84
Employed, yes, *n* (%)	46 (34.3)	18 (37.5)	18 (38.3)	10 (25.6)	0.40
Lives alone, yes, *n* (%)	25 (18.7)	8 (16.7)	10 (21.3)	7 (17.9)	0.85
Body mass index, kg/m^2^	29.3 ±4.8	29.8 ±5.1	29.3 ±4.5	28.6 ±4.8	0.58
Total complications, count	1.33 ±1.12	1.58 ±1.3	1.21 ±1.1	1.15 ±0.93	0.27
Total anti-diabetic medications, count	2.78 ±1.13	2.94 ±1.1	2.62 ±1.1	2.79 ±1.3	0.34
Systolic BP, mmHg	137.4 ±18.2	133.4 ±19.2	146.3 ±16.6	127.8 ±10.7	<0.01
Diastolic BP, mmHg	71.9 ±12.2	71.5 ±14.1	73.4 ±11.0	69.9 ±10.7	0.71
Total cholesterol, mg/dL	143.4 ±35.7	138.3 ±36.7	149.8 ±32.1	141.9 ±38.2	0.28
Triglycerides, mg/dL	159.8 ±72.2	167.0 ±17.4	167.4 ±97.0	141.7 ±77.4	0.57
HDL cholesterol, mg/dL	46.1 ±13.6	45.0 ±10.8	48.5 ±17.5	44.6 ±11.1	0.34
LDL calculated, mg/dL	68.7 ±25.0	65.9 ±28.8	72.5 ±28.8	67.3 ±29.8	0.54
Glucose, mg/dL	132.8 ±41.6	132.1 ±39.5	130.6 ±41.4	136.1 ±45.1	0.86
Hemoglobin A1C, %	7.1 ±1.1	6.9 ±1.1	7.3 ±1.2	7.1 ±0.8	0.26
Creatinine, mg/dL	1.0 ±0.5	1.2 ±0.8	0.9 ±0.3	0.8 ±0.3	0.01
Diabetes duration, years	19.1 ±9.7	20.6 ± 10.7	16.8 ±9.5	19.8 ± 8.5	0.15
Dietary knowledge score^b^, points	12.1 ±2.4	9.5 ±1.4	12.4 ±0.48	15.0 ±1.0	<0.01
MedD adherence^c^, points	11.0 ±2.1	10.7±1.8	11.0 ±2.3	11.3 ±2.3	0.48

Continuous variables are presented as mean ± SD. For categorical variables, Chi-square tests were used. For continuous variables, normality was assessed; normally distributed variables were compared using one-way ANOVA, while non-normally distributed variables were analyzed using the Kruskal–Wallis test.

*BP* blood pressure, *MedD* Mediterranean diet.

^a^Socioeconomic status ranges between 1 to 10.

^b^Knowledge scores can range from 0 to 17 points.

^c^MedD adherence score range is 0 to 17 points.

### Dietary knowledge

**Fig. 1:**
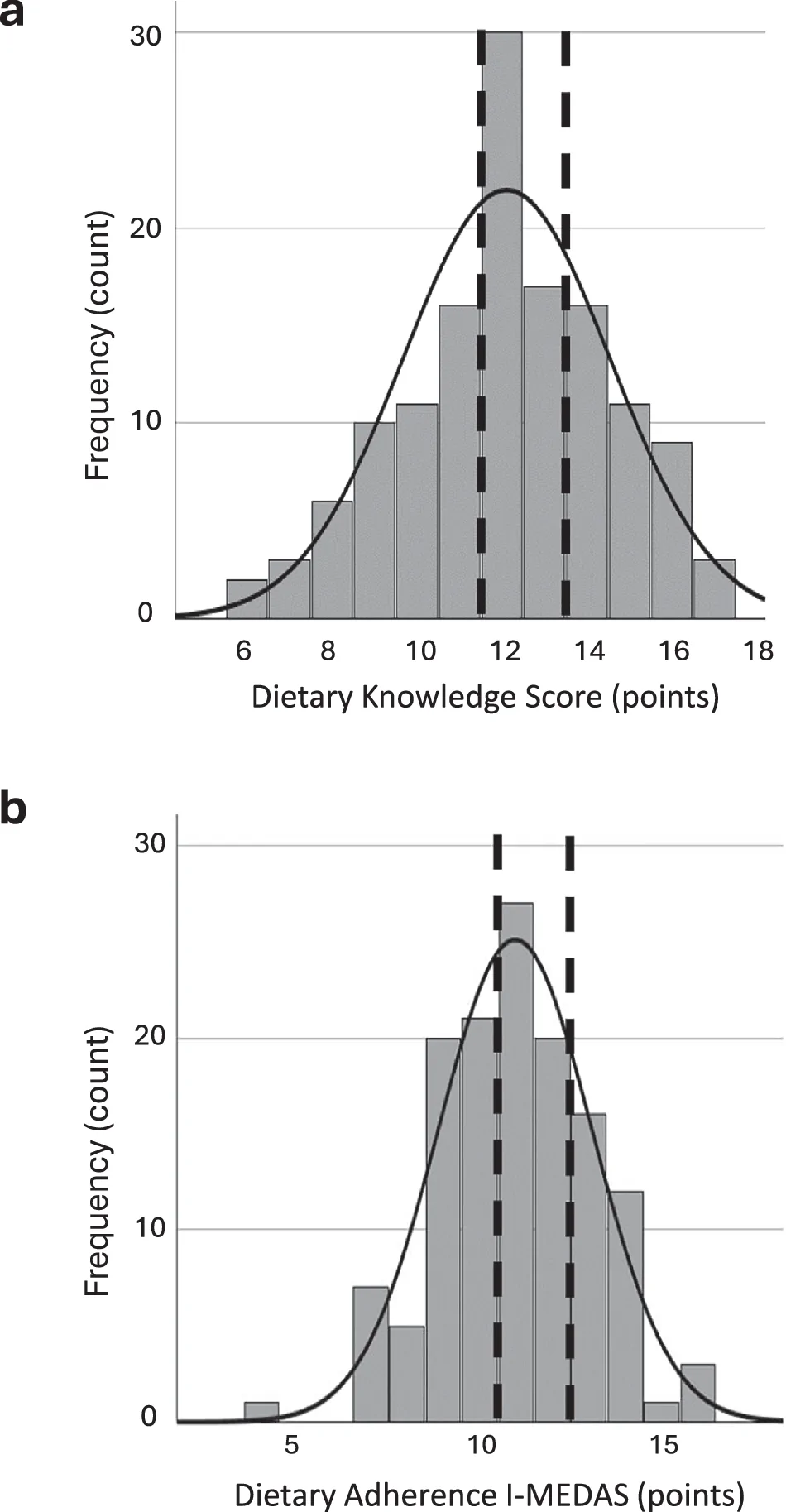
Distribution of dietary knowledge and Mediterranean diet adherence among study participants. **a** Distribution of dietary knowledge scores among the study population. *N* = 134. Mean ± SD = 12.1 ± 2.4. Score range 0–17 points. **b** Distribution of Mediterranean diet adherence scores based on the Israeli Mediterranean Diet Adherence Screener (I-MEDAS). *N* = 134. Mean ± SD = 11. 0 ± 2.1. Score range 0–17 points. Dotted lines indicate score tertiles.

The average dietary knowledge score was 12.1 ± 2.4, with a maximum score of 17 points. The scores were normally distributed (Fig. 1a). Three of the 17 questions of the dietary knowledge survey were answered incorrectly by at least half of the people. In these questions, most people thought that people with diabetes should strictly avoid certain foods, including grapes, salty pastries, and snacks, except for rare occasions. In contrast, the correct answer was that these items “can be consumed in moderation” (Table 2). In other words, for certain types of foods, people perceived the recommended diet for people with diabetes as more restrictive than necessary.

**Table 2:** Dietary knowledge scores, by sex.

	Correct response, *n* (%)			
Food item (The correct response^a^)	Overall	Women	Men	*p*-value
Coca Cola (Never)	129 (96.3)	70 (95.9)	59 (96.7)	0.80
Whole grains (Limited)	119 (88.8)	65 (89.0)	54 (88.5)	0.93
Olive oil or vegetable oil (Olive oil is better)	116 (86.6)	62 (84.9)	54 (88.5)	0.55
Olive oil (Limited)	112 (83.6)	62 (84.9)	50 (82.0)	0.65
Milk (Limited)	111 (82.8)	62 (84.9)	49 (80.3)	0.49
Packaged foods labeled for people with diabetes (Limited)	110 (82.1)	61 (83.6)	49 (80.3)	0.63
Nuts (Limited)	110 (82.1)	58 (79.5)	52 (85.2)	0.39
Butter (Limited)	107 (79.9)	62 (84.9)	45 (73.8)	0.11
Butter or margarine (Butter is better)	104 (77.6)	62 (84.9)	42 (68.9)	0.03
Mayonnaise or vegetable Oil (Oil is better)	93 (69.4)	53 (72.6)	40 (65.6)	0.38
Salt (Limited)	89 (66.4)	49 (67.1)	40 (65.6)	0.85
Watermelon (Limited)	87 (64.9)	51 (69.9)	36 (59.0)	0.19
Natural Juice 100% (Never)	85 (63.4)	50 (68.5)	35 (57.4)	0.19
White rice or white bread or pita (Limited)	73 (54.5)	42 (57.5)	31 (50.8)	0.44
Grapes (Limited)	67 (50.0)	42 (57.5)	25 (41.0)	0.06
Savory snacks (Limited)	59 (44.0)	31 (42.5)	28 (45.9)	0.69
Savory pastries (Limited)	51 (38.1)	27 (37.0)	24 (39.3)	0.78

Food items presented in the dietary knowledge questionnaire and the % of correct responses ranked from highest to lowest, and the difference in responses between men and women.

^a^The responses appeared in the dietary knowledge questionnaire as the following: “Can be consumed freely,” “Can be consumed in moderation” or “Can be consumed only in rare situations.” (Unlimited, Limited, Never, respectively).

In univariate analysis, people in the lowest tertile of dietary knowledge scores had higher serum creatinine levels compared to the other two tertiles. BMI, diabetes duration, and HbA1c were comparable across dietary knowledge tertiles. None of the other variables were significantly associated with the dietary knowledge score.

Women had slightly higher dietary knowledge scores as compared to men, 12.5 ± 2.4 vs 11.7 ± 2.4, respectively (*p* = 0.07). We explored whether men and women differed in their diabetes dietary knowledge regarding certain foods. Men and women had comparable rates of correct answers in all the food items in the dietary knowledge questionnaire, except that more men than women chose margarine over butter as the recommended fat for people with diabetes (31.1% vs 15.1%, *p* = 0.026; Table 2).

### MedD adherence

The MedD adherence scores were normally distributed (Fig. 1b), with an average score of 11.0 ± 2.1 points. Patients reported an especially low rate of adherence to certain components of the MedD (Table 3): only 18.8% reported consuming fish at least three times per week, 18.0% reported consuming legumes at least three times a week, and less than 50% of the people reported adequate consumption of nuts, tahini, hummus, and whole grain foods. On the other hand, more than 80% achieved the benchmark of good MedD adherence in terms of low consumption of sweet beverages, alcohol, savory snacks and pastries, frequent consumption of vegetables, and preferring poultry over meat. The MedD adherence scores of both sexes were comparable. Women had a higher rate of adherence compared to men in terms of low consumption of red meat (87.5% vs 68.9%, *p* < 0.01) and savory pastries (98.6% vs. 90.2%, *p* = 0.03); yet lower adherence to whole grain foods compared to men (34.7% vs. 52.5%, *p* = 0.04; Table 3).

**Table 3:** Adherence to specific foods included in the Israeli Mediterranean Diet Adherence Screener (I-MEDAS) questionnaire, by sex.

	Adherent response *n* (%)			
Screener item	Overall	Women	Men	*p*-value
Alcohol (≥7 servings/wk)	131 (98.5)	71 (98.6)	60 (98.4)	0.91
Salty snacks (<3 servings/wk)	130 (97.7)	70 (97.2)	60 (98.4)	0.66
Savory pastries (<2 servings/wk)	126 (94.7)	71 (98.6)	55 (90.2)	0.03
Sweet soft drinks (<1 servings/d)	126 (94.7)	70 (97.2)	56 (91.8)	0.17
Poultry more than red/processed meat (yes)	112 (84.2)	62 (84.9)	50 (82.0)	0.65
Non-starchy vegetables (≥2 servings/d)	110 (82.7)	59 (81.9)	51 (83.6)	0.80
Red/processed meat (<1 servings/d)	105 (78.9)	63 (87.5)	42 (68.9)	<0.01
Butter/margarine (<1 servings/d)	92 (69.2)	46 (63.9)	46 (75.4)	0.15
Desserts (<3 servings/wk)	91 (68.4)	49 (68.1)	42 (68.9)	0.92
Fruit (≥3 servings/d)	81 (60.9)	47 (65.3)	34 (55.7)	0.27
Non-sweetened dairies (≥2 servings/d)	78 (58.6)	45 (62.5)	33 (54.1)	0.33
Olive oil is my main dietary oil (yes)	75 (56.4)	38 (52.1)	37 (60.7)	0.32
Whole grain (≥3 servings/d)	57 (42.9)	25 (34.7)	32 (52.5)	0.04
Nuts (≥3 servings/wk)	49 (36.8)	28 (38.9)	21 (34.4)	0.60
Hummus or tahina (≥3 servings/wk)	47 (35.3)	21 (29.2)	26 (42.6)	0.11
Fish (≥3 servings/wk)	25 (18.8)	13 (18.1)	12 (19.7)	0.81
Legumes (≥3 servings/wk)	24 (18.0)	9 (12.5)	15 (24.6)	0.07

The defined key for each point appears in parenthesis.

*wk* week, *d* day.

### The association between dietary knowledge and MedD adherence

**Fig. 2:**
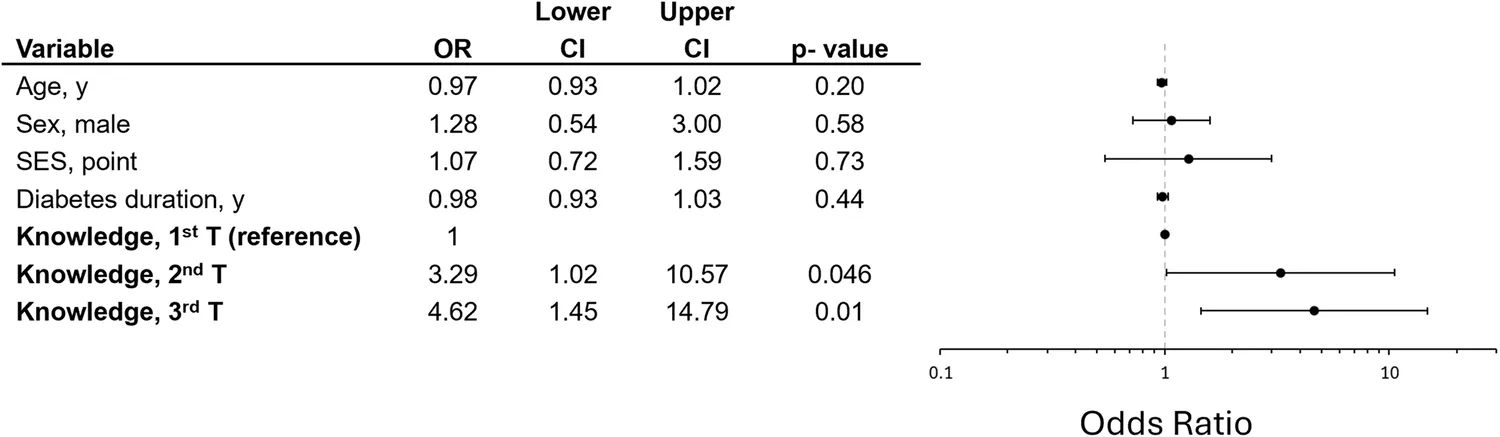
Mediterranean diet adherence, multivariate analysis. The association between dietary knowledge and Mediterranean diet adherence using multivariate logistic regression. Mediterranean diet adherence was defined as positive when Israeli Mediterranean Diet Adherence Screener (I-MEDAS) score was in the upper tertile. Age and diabetes duration odds ratios are per 1 year. SES odds ratio for each point. OR odds ratio, CI 95% confidence interval, SES socio-economic state, T tertile.

The unadjusted association between dietary knowledge and MedD adherence assessed by chi-square test showed a trend toward positive correlation (*p* = 0.06, Table 4). Following our pre-specified analytical plan, we proceeded with multivariable logistic regression adjusted for age, sex, diabetes duration and socio-economic status. In this primary analysis, people with dietary knowledge scores in the second or third tertiles were significantly more likely to be in the highest tertile of MedD adherence compared to the lowest tertile of dietary knowledge (OR = 3.29, 95% CI 1.02–10.57, *p* = 0.046; and OR = 4.62, 95% CI 1.45–14.79, *p* = 0.01, respectively; Fig. 2). The model explained limited variance in adherence (Nagelkerke R² = 0.16), indicating additional unmeasured factors likely contribute to MedD adherence patterns. Two sensitivity analyses were performed. When adding clinical variables (BMI ≥ 30 kg/m^2^, HbA1c, number of antidiabetic medications and number of diabetic complications) to the primary logistic regression model, the association persisted (OR for third tertile of dietary knowledge = 6.31, 95% CI 1.69–23.60, *p* = 0.003). When defining dietary knowledge and MedD adherence scores as continuous variables and applying a multivariate linear regression model, the association was not statistically significant (standardized coefficient Beta = 0.17, *p* = 0.09).

**Table 4:** The unadjusted association between dietary knowledge and Mediterranean diet adherence scores.

		Dietary knowledge score, tertiles			
		T1, *n*	T2, *n*	T3, *n*	Total
Dietary adherence score, tertiles	T1, *n*	21	18	15	54
	% within adherence	38.9	33.3	27.8	100.0
	% within knowledge	44.7	38.3	38.5	40.6
	T2, *n*	21	16	10	47
	% within adherence	44.7	34.0	21.3	100.0
	% within knowledge	44.7	34.0	25.6	35.3
	T3, *n*	5	13	14	32
	% within adherence	15.6	40.6	43.8	100.0
	% within knowledge	10.6	27.7	35.9	24.1
	Total	47	47	39	133
	% within adherence	35.3	35.3	29.3	100.0
	% within knowledge	100.0	100.0	100.0	100.0

Univariate analysis of the association between nutritional knowledge and dietary adherence. Knowledge and adherence are presented according to score tertiles. The analysis included 133 people who had completed both questionnaires. Chi-square test, *p* = 0.06.

## Discussion

This study investigated the relationship between dietary knowledge and MedD adherence in people with T2D in Israel. After controlling sociodemographic factors, higher dietary knowledge was associated with higher MedD adherence score, with those in the highest knowledge tertile showing higher odds of adherence compared to those with lowest tertile of knowledge. However, this association showed limited robustness: the model explained only 16% of variance in adherence, the wide confidence interval indicated substantial uncertainty about the magnitude of effect, and in sensitivity analysis defining the exposure and outcome as continuous variables the association was not statistically significant. These findings suggest that dietary knowledge, along with measured sociodemographic factors, explains only a modest proportion of MedD adherence variability in this population.

A few studies have specifically focused on diabetes-related dietary knowledge and adherence to diabetes nutrition. In a meta-analysis of observational studies conducted among people with diabetes in Ethiopia, dietary knowledge was a strong predictor of dietary adherence (pooled OR: 2.2; 95% CI: 1.6–2.8). The overall proportion of people included in the meta-analysis, who were dietary adherent, was relatively low (41%) [27]. Cross-sectional studies in Ghana [28] and Sudan [29] also supported an association between diabetes-specific dietary knowledge and dietary adherence in people with T2D. However, in a small study conducted in Singapore, dietary knowledge among people with T2D was relatively poor, and its correlation with dietary adherence was not statistically significant (Pearson’s correlation r = –0.29; *p* = 0.065) [30]. Similarly, among Thai participants, diabetes dietary knowledge did not significantly correlate with dietary adherence, as measured by macronutrient composition of reported food intake [31]. We did not find published data on the association between dietary knowledge and adherence in Middle Eastern populations. Our findings align with those studies, showing weak or non-significant associations. The wide confidence interval and limited explained variance, combined with loss of statistical significance in sensitivity analyses, indicate that the knowledge-adherence relationship in our population is modest and sensitive to analytical approach.

In the current study, we found a relatively high adherence to the local version of the MedD among people with diabetes, as measured by the I-MEDAS questionnaire, with a mean score of 11.0 ± 2.1 (range 0–17). In comparison, the median score of the I-MEDAS questionnaire among adults in the general population in Israel was lower (8, IQR: 7–9) [23]. These differences could be related to the fact that the population in the current study had an older age, higher socio-economic background, and long-standing diabetes, and therefore, is more likely to have higher health literacy, prior consultation with a dietitian, motivation, and availability of resources, which may all lead to higher nutritional adherence.

The limited explanatory power of our model (Nagelkerke R² = 0.16) indicates that dietary knowledge and basic sociodemographic factors capture only a small fraction of the variance in MedD adherence, which was also reported by others [32, 33]. This large unexplained variability suggests that other factors affecting dietary adherence among people with T2D were unaccounted for in our model. Several categories of unmeasured factors may explain this variability. These may include clinical factors such as a recent admission or the new onset of a diabetes-related complication, which can affect patient compliance, including dietary adherence; behavioral and psychological factors, such as meal planning and food preparation skills, patient health literacy and beliefs [34] and inhibitory control [32]; and environmental and social factors such as individual-household income and food prices, which have been shown to influence adherence to healthful diets [35, 36]. Other studies have found that cognitive performance was not associated with dietary adherence [37].

These findings have important clinical implications: In populations with long-standing diabetes, high health literacy, and good care access, knowledge-based interventions alone may have limited impact on dietary adherence. Diabetes educators and dietitians should assess and address other modifiable factors beyond dietary knowledge—including behavioral skills, motivation, self-efficacy, and environmental barriers—in their nutrition counseling. This is indeed recommended by current diabetes management guidelines [7], yet multi-component nutrition interventions remain understudied in routine clinical practice settings, and evidence for their effectiveness in improving long-term dietary adherence is limited [1, 38]. Additional research is needed to determine whether systematically targeting these factors translates to improved long-term dietary adherence and clinical outcomes.

This study has several limitations. First, the cross-sectional design prevents us from determining causality in the knowledge-adherence relationship. Second, participants were recruited from a single tertiary diabetes center using convenience sampling. The study population was relatively homogeneous (96% Jewish, mean SES 7.8/10), with low representation of younger adults, lower socioeconomic status, and Arab ethnicity, limiting generalizability to a wider range of the diabetes population in Israel. Most patients’ cardiovascular risk factors were well-controlled, suggesting a selective, health-engaged subgroup rather than the broader diabetes population. Third, although the dietary knowledge questionnaire underwent face and content validity assessment, full psychometric validation was not conducted, as this was a cultural adaptation of an existing validated instrument rather than de novo scale development. Internal consistency was modest (Cronbach’s α = 0.52), yet comparable to other validated dietary knowledge instruments [39, 40]. Fourth, the questionnaire did not assess all aspects of diabetes nutrition knowledge, such as meal planning, label interpretation, or portion sizing. Finally, adherence was measured through self-report, which introduces potential for recall and social desirability bias.

In conclusion, this cross-sectional study demonstrated a weak positive association between dietary knowledge and MedD adherence in people with T2D from a tertiary diabetes clinic in Israel. The substantial unexplained variability highlights the need to investigate other potentially modifiable factors beyond nutrition knowledge – such as behavioral skills, self-efficacy, and food environment – that may play a more substantial role in determining dietary adherence. These findings warrant validation in larger, more diverse populations. Additional research is needed to determine whether multi-component interventions systematically targeting these factors can improve long-term dietary adherence and clinical outcomes.

## Data Availability

Data are not publicly available because this is not covered by the informed consent of participants. However, access to the data in the local database is possible upon reasonable request and according to approval of the local ethics committee. Further inquiries can be directed to the corresponding author.
